# Physiological Effect of Ghrelin on Body Systems

**DOI:** 10.1155/2020/1385138

**Published:** 2020-05-25

**Authors:** Yonas Akalu, Meseret Derbew Molla, Gashaw Dessie, Birhanu Ayelign

**Affiliations:** ^1^Department of Physiology, School of Medicine, College of Medicine and Health Sciences, University of Gondar, Gondar, Ethiopia; ^2^Department of Biochemistry, School of Medicine, College of Medicine and Health Sciences, University of Gondar, Gondar, Ethiopia; ^3^Department of Immunology and Molecular Biology, School of Biomedical and Laboratory Science, College of Medicine and Health Sciences, University of Gondar, Gondar, Ethiopia

## Abstract

Ghrelin is a relatively novel multifaceted hormone that has been found to exert a plethora of physiological effects. In this review, we found/confirmed that ghrelin has effect on all body systems. It induces appetite; promotes the use of carbohydrates as a source of fuel while sparing fat; inhibits lipid oxidation and promotes lipogenesis; stimulates the gastric acid secretion and motility; improves cardiac performance; decreases blood pressure; and protects the kidneys, heart, and brain. Ghrelin is important for learning, memory, cognition, reward, sleep, taste sensation, olfaction, and sniffing. It has sympatholytic, analgesic, antimicrobial, antifibrotic, and osteogenic effects. Moreover, ghrelin makes the skeletal muscle more excitable and stimulates its regeneration following injury; delays puberty; promotes fetal lung development; decreases thyroid hormone and testosterone; stimulates release of growth hormone, prolactin, glucagon, adrenocorticotropic hormone, cortisol, vasopressin, and oxytocin; inhibits insulin release; and promotes wound healing. Ghrelin protects the body by different mechanisms including inhibition of unwanted inflammation and induction of autophagy. Having a clear understanding of the ghrelin effect in each system has therapeutic implications. Future studies are necessary to elucidate the molecular mechanisms of ghrelin actions as well as its application as a GHSR agonist to treat most common diseases in each system without any paradoxical outcomes on the other systems.

## 1. Introduction

Ghrelin, a unique 28-amino-acid peptide, is the first identified circulating hunger hormone. It is a hormone in the endocrine system and a neurotransmitter in the nervous system. It is also called growth hormone secretagogue or motilin-related peptide [1]. It was discovered in 1999 by Kojima in Japan after the discovery of the growth hormone secretagogue type 1a receptor (GHSR1a), in 1996 [2]. The name is based on its role as a growth hormone-releasing peptide, concerning the Proto-Indo-European root ghre, meaning to grow (*G*rowth *H*ormone *Rel*easing *In*ducing = ghrelin) [3].

**Figure 1:**
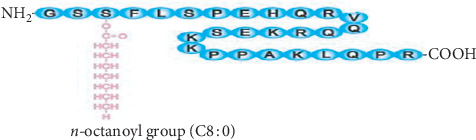
The structure of ghrelin. Ghrelin is a 28-amino-acid peptide with an n-octanoyl group attached to serine at position 3 that is critical for the majority of its activity.

Ghrelin has two forms: acyl ghrelin (octanoylated form) and des-acyl ghrelin (nonoctanoylated form). The octanoylation of ghrelin is critical for its physiological functions which depend upon ghrelin O-acyltransferase (GOAT) catalyzation, and 20% of ghrelin is found as octanoylated at the third carbon (Figure 1) [4, 5]. Des-acyl ghrelin is a nonoctanoylated and inactive form that does not activate the growth hormone secretagogue receptor, which is a target for acyl form to release growth hormone (GH). Des-acyl ghrelin is now known to have independent physiological functionality [6]. Receptors (GHSR) 1a and 1b, encoded by a gene located on 3q26.31, are widespread and are found in many parts of the body and even in tumors and metastases [1, 7]. Ghrelin and its receptors are widely expressed in many regions of the brain [8, 9], pituitary [10], intestine [11], kidney [8], thyroid gland [12, 13], lung [8, 14], heart [11, 15], pancreatic islets [16], ovaries [8], testis [17], and sebaceous glands [18, 19]. GHSR1a is expressed mainly in the anterior pituitary gland, pancreatic islets, adrenal gland, thyroid, myocardium, arcuate nucleus, hippocampus, the substantia nigra pars compacta, ventral tegmental area (VTA), raphe nuclei [18, 20, 21], cortex, and parafascicular thalamic region [22]. This wide distribution of ghrelin receptors implies its broad physiological effect. Ghrelin has a plethora of functions. This review aims to discuss and explain the physiological effects of ghrelin in the body using a systemic approach. This, in turn, attracts researchers to emphasize it and to prepare different therapy to treat diseases in each respective body part.

### 1.1. Regulation of Ghrelin Production

Ghrelin is produced by X/A-like cells in rats and P/D1 cells in humans which are found distributed throughout the mucosa of the stomach [23, 24]. This discrete type of stomach cells is the major source of circulating ghrelin [25, 26]. The secretion, degradation, and clearance rates of ghrelin determine its blood level. Acyl ghrelin is des-acylated by plasma esterases and the circulating ghrelin is captured by its receptor, degraded by plasma proteases, and excreted in urine [27, 28]. Ghrelin synthesis and secretion are affected by different conditions such as fasting and pathological conditions [28]. Plasma ghrelin levels increase during fasting and decrease in feeding [29]. The mechanism of increase in ghrelin levels during fasting is evidenced to be noradrenergic mediated [30] and the postprandial decrease by an increase in glucose and insulin [30, 31]. Fasting increases gastric ghrelin mRNA expression in mice and rats [32, 33]. Expression and secretion of ghrelin are inversely associated with the gastric mechanistic target of rapamycin (mTOR) signaling [34]. Knockout of mTOR in X/A-like cells increases circulating acyl ghrelin. Removal of mTOR upstream inhibitor, tuberous sclerosis 1, activates its signaling and decreases ghrelin expression and secretion. mTOR is an intracellular energy sensor [35, 36] and its activity is regulated by nutrients, energy supply, and various hormones [37, 38].

Chronic intake of high-calorie diets, prolonged exposure to high-fat, and obesity result in a reduction of stomach production and secretion of ghrelin [29, 39]. However, an increase in the number of ghrelin-secreting cells in response to the high-fat diet has been shown in another study [40]. The extent to which the increased adiposity exerts an inhibitory influence on stomach ghrelin production and secretion is not well known [41].

Ghrelin release is also modulated by different factors, such as peptide hormones, monoaminergic neurotransmitters, glucose, fatty acids, second messengers, and potential downstream effector enzymes and channels. Glucose, long-chain fatty acids, insulin, glucagon, GH inhibitory hormone, oxytocin, and dopamine (DA) regulate ghrelin release by directly acting on ghrelin-producing cells [30, 42–44]. Glucose or amino acids have been found to suppress ghrelin levels more rapidly and effectively than lipid infusions. This may be due to the difference in absorption rate of glucose and amino acids, which are quickly absorbed from the gut, suppressed ghrelin rapidly and deeply, while lipids that require intestinal digestion before absorption lead to weak suppression of ghrelin levels [45]. The possible mechanism for suppression of ghrelin production by food may be due to the capability of ghrelin-producing cells to sense nutrients directly or gut hormones (insulin, glucagon-like-peptide 1 (GLP-1), peptide YY (PYY), and cholecystokinin (CCK)) produced following a meal [27]. Numerous observations in humans indicate that insulin may inhibit ghrelin secretion. GLP-1 has been reported to lessen the preprandial rise of ghrelin in human beings by stimulating insulin secretion [46]. The inhibitory effect of glucose on ghrelin release may be mediated by the stimulatory effect of glucose on insulin [47]. Glucagon may directly stimulate the gene transcription of ghrelin [48]. It has been shown that leptin inhibits both the secretion of gastric ghrelin and the stimulation of ghrelin-induced feeding [49]. GH exerts a negative feedback action on ghrelin production and secretion [50]. Insulin-like growth factor-1(IGF-1) induces ghrelin secretion directly or indirectly by inhibiting GH secretion [51]. Cortisol and fatty acids also exert negative feedback on ghrelin secretion [52].

The autonomic nervous system is also one of the main regulators of ghrelin production. Ghrelin secretion is modulated by the balance between cholinergic and adrenergic tones that control the enteric nervous system [53, 54]. A study in rats and humans shows that plasma ghrelin increases following administration of muscarinic agonists and decreases following administration of muscarinic antagonist [55, 56]. Plasma acyl ghrelin concentration is increased by the *α*-adrenergic antagonists and *β*-adrenergic agonists that act directly on *β*1 receptors in ghrelin-secreting cells [30]. Excitation of the vagus nerve and the enteric nervous system in the stomach mucosa directly stimulates ghrelin-producing cells [28]. Understanding the mechanisms of ghrelin regulation is important for better therapeutic benefits of ghrelin, by modifying its secretion, acylation, and degradation. Furthermore, it is also important for preparing ghrelin agonists and antagonists.

## 2. Methodology

### 2.1. Type of Review

Narrative literature review was conducted.

### 2.2. Inclusion and Exclusion Criteria

Studies on animal (rats or mice) models and human trials published in English language were included. Reviews and other nonexperimental studies were also included. There was no restriction on publication year; studies from the discovery of ghrelin, 1999, to January 2020 were included. Studies conducted on fish, birds, and animals other than rat/mouse models and human trials were excluded from the review. Citations without abstract and/or full text were also excluded from the review.

### 2.3. Sources and Searching Strategies

PubMed, Google Scholar, and direct Google search were performed to find out all publications describing the effect of ghrelin on each body system and body part. The basic search terms and phrases were “ghrelin,” “acyl ghrelin,” “des-acyl ghrelin,” “growth hormone secretagogue,” “motilin-related peptide,” “effect,” “action,” and “role”. Names of all body systems, major body parts, and basic physiological processes carried out in each body part and system were used as search terms and phrases. In our searching strategy, we included a combination of words or phrases interrelated to the effect of ghrelin on different parts of the body. We screened the reference lists of reviews to locate additional primary studies that were not picked up by our search. To avoid missing studies in the search strategy, we considered different terms and names. The search strategy with the PubMed database was (“ghrelin” OR “acyl ghrelin” OR “des-acyl ghrelin” OR “growth hormone secretagogue” OR “motilin-related peptide”) AND (“physiological effect” OR “role” OR “action”).

### 2.4. Selection of Studies

To remove duplicate studies, all the repossessed studies were exported to Endnote version 7. Two independent reviewers (YA and MDM) screened the titles and abstracts. Disagreement between the two reviewers was handled by a third reviewer (BA) based on conventional article selection criteria.

### 2.5. Quality Assessment

All papers selected for inclusion in the review were appraised by independent investigators using a *scale* for the *assessment* of nonsystematic review articles (*SANRA*) [57]. This scale has six items that are rated in integers from 0 (low standard) to 2 (high standard), with 1 as an intermediate score. The possible maximal sum score is 12.

**Figure 2:**
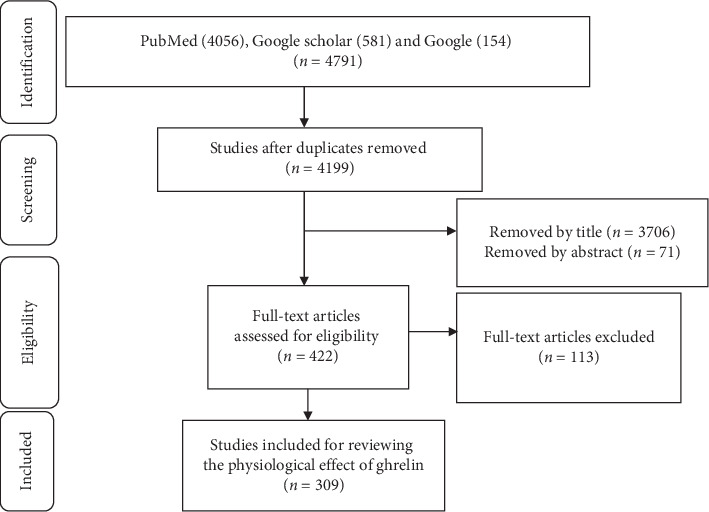
Study Selection process using PRISMA.

After full searching, we retrieved 4056 studies from PubMed, 540 studies from Google Scholar, and 95 studies from Google. Five hundred ninety-two studies were removed due to duplication using Endnote 7. From the screened studies, 3777 studies were eliminated after reviewing the titles and abstracts, and 110 studies were removed after reviewing the full text. A total of 212 studies were included in the review (Figure 2). We reviewed these studies and found that ghrelin affects all body systems. Thus, it is difficult to suggest future researchers to concentrate on a specific system. Similarly, recommending future researchers to concentrate on all systems is vague. Therefore, we recommend future researchers to concentrate not on all diseases in each system, but on the most common diseases like cardiovascular disease or metabolic diseases.

## 3. System-Based Understanding of Physiological Effect of Ghrelin

**Figure 3:**
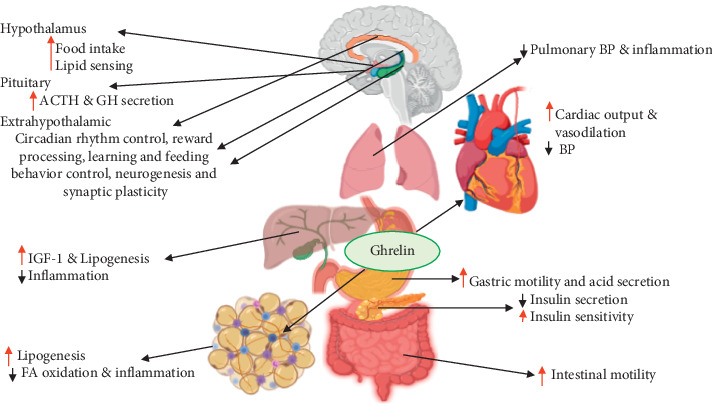
Physiological effect of ghrelin. ACTH: adrenocorticotropic hormone; IGF-1: insulin-like growth factor-1; BP: blood pressure. Up/down arrows denote increase/decrease.

The most prominent and early known effects of ghrelin following its discovery are appetite stimulation and GH release stimulation [7, 58, 59]. Nowadays, different studies showed that ghrelin has many functions beyond the initial discoveries (Figure 3). The physiological effect of ghrelin on each system is explained as follows.

### 3.1. Effect of Ghrelin on Nervous System

**Figure 4:**
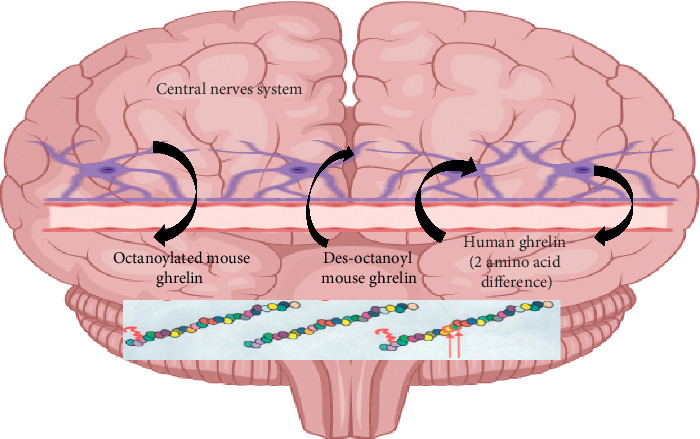
Transport of h-ghrelin, m-ghrelin, and des-m-ghrelin across the BBB of mice. Although octanoylated (bioactive) m-ghrelin crosses the mouse BBB predominantly in the brain-to-blood direction, passage for des-m-ghrelin was observed only in the blood-to-brain direction. H-ghrelin, which differs from m-ghrelin in two amino residues only, was transported in both directions in mice. The extent to which and the direction in which the ghrelin can cross the BBB are therefore influenced by at least two features of its primary structure, its posttranslationally added fatty acid side chain and its amino acid sequence.

Ghrelin has been known to have an effect on many parts of the brain mainly on the hypothalamus [60]. Even though the hypothalamus is the main target of ghrelin to regulate appetite and other endocrine functions, stimulation of GHSR on other areas of the brain like caudal brainstem and third and fourth ventricles significantly increased appetite and food intake [61]. Ghrelin mediates multiple physiological functions beyond those involved in metabolic activity. However, brain areas, other than circumventricular organs, which are a target for ghrelin, are protected by the blood-brain barrier (BBB). It is known that peptide and protein hormones could not cross the BBB. Therefore, ghrelin often requires saturable specialized transporters to cross the BBB [62]. To understand this, three forms of radioactively labeled ghrelin peptide were tested on mouse BBB: human ghrelin (h-ghrelin), mouse ghrelin (m-ghrelin), and des-octanoyl mouse ghrelin (des-m-ghrelin) [63]. It was found that each of these molecules crossed the BBB but differed in degree of passage, direction of passage, and transport mechanism. Direction and extent of passage are determined by the primary structure of ghrelin, defining a new role for the unique posttranslational octanoylation [64]. Des-acyl ghrelin travels into the brain from the blood easily via nonsaturable diffusion, whereas h-ghrelin was readily transported by a saturable system in both directions, across the BBB. Moreover, h-ghrelin also demonstrates both saturable binding and endocytosis in in vitro studies using rat cerebral microvessel endothelial cells [65]. Transport of m-ghrelin was saturable only from the brain to the blood direction. M-ghrelin differs from h-ghrelin in two of its 28 residues, with lysine replacing arginine at position 11 and alanine replacing valine at position 122. These two amino acids are, therefore, critical for recognition by the blood-to-brain transporter but not the brain-to-blood transporter. Acyl ghrelin is incapable of crossing lipid bilayers unaided [63]. Saturable transport system directed in the brain-to-blood direction, which has a similar affinity for m-ghrelin and h-ghrelin, requires the presence of the unique octanoyl component of the ghrelin molecule [66] (Figure 4).

**Figure 5:**
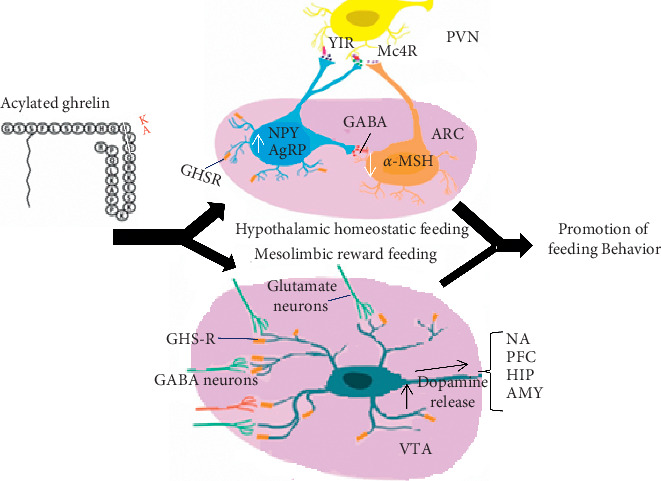
Effect of ghrelin on two main brain regions: arcuate nucleus (ARC) of the hypothalamus and the ventral tegmental area (VTA). The h-ghrelin is represented in the figure as a black amino acid sequence, and red-letter substitution is that of the rat. Acyl ghrelin is proposed to initiate neurocircuits that promote feeding behavior in the ARC and VTA. Within the ARC, ghrelin stimulates neuropeptide Y/agouti-related peptide (NPY/AGRP) neurons by binding to GHSR on their surface. Upon activation, these neurons produce and release *γ*-aminobutyric acid which inhibits anorectic proopiomelanocortin (POMC) neurons, decreasing the release of the anorectic peptide *α*-melanocyte-stimulating hormone (*α*-MSH). This efficiently reduces the amount of *α*-MSH capable of binding to satiety promoting melanocortin 4 receptors (MC4Rs). Simultaneously, activated NPY/AGRP neurons increase their production and secretion of orexigenic peptides NPY and AGRP. NPY binds to neuropeptide Y receptor type 1 (Y1R), and AGRP antagonizes the binding of *α*-MSH at MC4Rs. These two effects, the reduction in anorectic and enhancement of orexigenic peptides, work to reduce the activity of second-order anorexigenic neurons in the paraventricular nucleus (PVN) to promote homeostatic feeding behavior [66]. Similarly, ghrelin also stimulates VTA DA neurons, increasing the frequency and probability of the DA release from their projections in the nucleus accumbens (NAcc), prefrontal cortex (PFC), hippocampus, and amygdala to encourage mesolimbic reward feeding [66].

Once ghrelin gets access to different regions of the brain, it promotes feeding (Figure 5) and has also an effect on higher brain functions, motor functions, and sensory functions.

#### 3.1.1. Effect of Ghrelin on Higher Brain Functions

**Figure 6:**
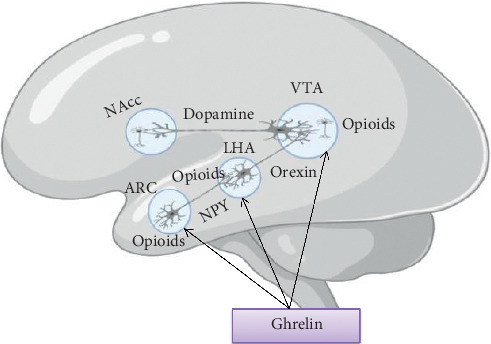
Brain pathways mediating ghrelin's effects on food motivation. The main pathway involved in food motivation is the midbrain DA projection from the VTA to the NAcc. Ghrelin appears to activate this pathway directly at the level of the VTA, which in turn gives a potential mechanism for ghrelin to promote food intake even when homeostatic hypothalamic centers such as the ARC or lateral hypothalamic area (LHA) indicate a state of satiety. Ghrelin also affects food motivation indirectly by activating an afferent pathway. At the level of the VTA, opioid signaling (but not NPY signaling) is required for ghrelin's effects on food motivation.

GHSR is widely expressed in the brain regions like the hippocampus [7] and other areas controlling emotional responses [67], memory, and learning [68]. Numerous studies showed that ghrelin can regulate numerous higher brain functions including learning memory and reward-seeking behavior [22, 42, 67, 69–71]. Ghrelin activates the reward system, namely, the mesolimbic DA system [72] (Figure 6). Ghrelin is also important for sleep regulation [22, 73], and a study in healthy men revealed that ghrelin promotes slow-wave sleep [74]. This is a good insight to treat patients with poor sleep quality.

*(1) Effect of Ghrelin on Reward Neurocircuits*. Central reward pathways, which encompass multiple interconnected brain regions, are sensitive to hormonal peptides like ghrelin involved in the control of feeding [75]. The ghrelin receptor is localized in many brain regions associated with pleasure, reward, and motivation [20]. Ghrelin signaling provides a strong driving force to ensure both an adequate nutritional supply and a diversity of food of varying reward value [66]. The most known reward pathway is the dopaminergic innervation of the NAcc by neurons in VTA. Other regions, especially the target sites of the NAcc (such as the prefrontal cortex and ventral pallidum) and inputs into the NAcc (such as the amygdala), are also important for reward processing. Other regions implicated in ghrelin signaling such as the lateral and ventromedial hypothalamus and the arcuate nucleus also form neurocircuits with the reward pathways that are likely to be important in integrating feeding control [76].

Ghrelin stimulates VTA DA neurons both directly by binding to GHSR receptors located on their surface and indirectly by increasing the ratio of excitatory to inhibitory synapses. This stimulation increases the frequency and probability of DA release from their projections in the NAcc, PFC, HIP, and amygdala to encourage mesolimbic reward feeding [66, 77] (Figure 6). Suppression of ghrelin signaling interrupts reward from chemical drugs of abuse such as alcohol [78], nicotine [79], cocaine, and amphetamine [80]. This is good news for treating patients who suffer from substance abuse.

**Figure 7:**
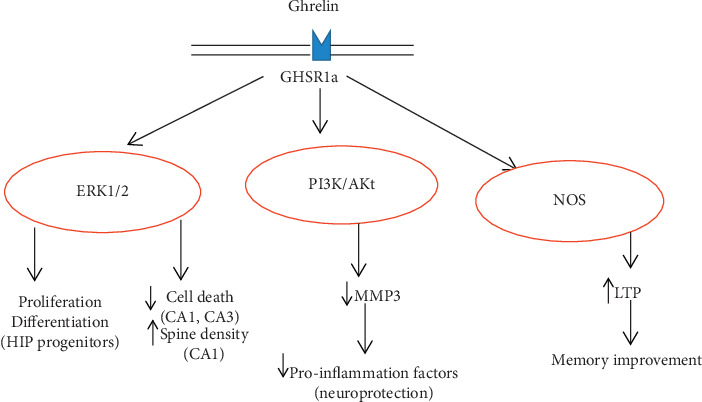
Representation of assumed mechanistic pathways involving ghrelin and its respective receptors in hippocampal cells. The intracellular pathways leading to functional outcomes related to memory modulation are shown in red. HIP: hippocampus; MMP: matrix metalloproteinase; LTP: long-term potentiation; ERK: extracellular signal-regulated kinase; CREB: cAMP response element-binding protein; NOS: nitric oxide synthase; PI3K/Akt: phosphatidylinositol 3-kinase and protein kinase B [70].

*(2) Effect of Ghrelin on Memory and Learning*. The main brain sites responsible for memory and learning are the hippocampus and parahippocampal formation, comprising the entorhinal and perirhinal cortices. These areas have a significant role in the acquisition of new memories and long-term memory retention [81, 82]. Ghrelin acts on numerous sites in the hippocampus mostly on the dentate gyrus and the Cornu Ammonis (CA) regions, CA1 and CA3 [83], to improve memory through its neuroprotective action. It influences pathways involved in neuronal plasticity, which is important for memory [84]. Chronic treatment with intraperitoneal ghrelin increases synaptic dendritic spines density in the CA1 region of mice [83]. Ghrelin also induces long-term potentiation by both pre-and postsynaptic mechanisms [75] on hippocampal slice preparations [75]. Ghrelin inhibits cell death by activating the phosphatidylinositol 3-kinase and protein kinase B (PI3K/Akt) pathway in the hippocampus resulting in improvement of memory [85]. Intrahippocampal ghrelin increases nitric oxide synthase (NOs) to induce short-term and long-term memory improvement [86] (Figure 7). The lower level of fasting ghrelin and inhibition of ghrelin signaling in humans were associated with memory loss and cognitive impairment [87, 88], even though other studies showed that ghrelin has no effect on memory; instead, it modulates encoding-related brain functions without enhancing memory formation [89].

#### 3.1.2. Sensory Function

Ghrelin has also an effect on the sensory function of the nervous system. It has been reported to have analgesic effects on peripheral pain in the rat model which was mediated via transient receptor potential vanilloid type I opioid systems [90]. A recent study done on mice revealed that injection of ghrelin initially activated the GHSR1a, which in turn increased the release of endogenous proenkephalin for activation of the *δ*-opioid receptor to produce antinociception [91]. Ghrelin also exerts analgesic effects on inflammatory pain through modulation of IL-10 and TGF-*β* levels in a rat model [92]. Moreover, ghrelin is also important for a taste sensation, olfaction, and sniffing [42]. Ghrelin renders the olfactory system more responsive to odors [93].

#### 3.1.3. Motor Function

The presence of GHSR1a throughout the dopaminergic pathway and low level of ghrelin in Parkinson's disease, characterized by low DA, indicate that ghrelin has a role in DA signaling or dopaminergic pathway [94]. Intraperitoneal injections of acyl ghrelin in mice model protect substantia nigra dopaminergic neurons against neuronal death [42, 95, 96]. Interaction of ghrelin and DA in the mesocorticolimbic system controls reward-based locomotor activity [97]. In the mesolimbic dopaminergic pathway, central ghrelin administration to the third ventricle induced an acute increase in locomotor activity as well as DA-overflow in the NAcc, which could be antagonized by the GHSR1a antagonist [79, 98]. Ghrelin was found to antagonize both the dopaminergic neuron loss in the substantia nigra pars compacta and the depletion of DA levels in the striatum [22].

*(1) Effect of Ghrelin on Autonomic Nervous System*. Different studies showed that ghrelin inhibits sympathetic nervous activity. Intravenous (IV) injection of ghrelin to the rat developing sepsis significantly reduced the elevated norepinephrine (NE) and tumor necrosis factor-alpha (TNF-*α*) level. Administration of GHSR1a antagonist significantly increased NE and TNF-*α* levels [99]. This implies the inhibitory effect of ghrelin on the sympathetic nervous system. The same result was reported by a study done on healthy volunteers. Single administration of ghrelin decreased both heart rate and blood pressure [100]. This effect gives insight into the preparation of antihypertensive drugs from ghrelin.

#### 3.1.4. The Role of Ghrelin in CNS Pathologies

Ghrelin influences several pathways involved in inflammation, neurogenesis, and apoptosis. Neurogenesis, including the proliferation, migration, and differentiation of neurons, occurs mainly in the dentate gyrus [84]. Ghrelin activates cellular proliferation in the subventricular zone by acting on GHSR1a and other types of receptors [101]. Ghrelin acts as a potent growth factor to stimulate cell proliferation and acts directly on rat dorsal motor nucleus of the vagus neurons, which contains ghrelin receptor, to stimulate neural proliferation and neurogenesis in vivo and in vitro [102]. Ghrelin stimulates cellular differentiation and proliferation and exerts cell-protective effects in adult rat hippocampal progenitor cells [101, 103]. Due to its antiapoptotic and anti-inflammatory effect, ghrelin preserves the normal function of cortical neurons following epileptic seizures by reducing necrosis and loss of nerve through significantly inhibiting mRNA expression of proinflammatory molecules like TNF-*α*, IL-1*β*, and cyclooxygenase 2 [97, 104, 105]. Exogenous des-acyl ghrelin protected the brain from ischemia and hypoxia via eliciting a powerful vasodilator response on cerebral arteries. Also, exogenous des-acyl ghrelin suppressed superoxide production in cerebral arteries [106]. A recent study shows that ghrelin attenuates secondary brain injury following intracerebral hemorrhage by inhibition of nucleotide-binding oligomerization domain-like receptor pyrin domain-containing 3 (NLRP3) inflammasome activation, and promotion of nuclear factor E2-related factor 2 (Nrf2) antioxidative response element signaling pathway [107]. Similarly, ghrelin protects adult rat hypothalamic neuronal cells from apoptosis and excessive autophagy due to oxygen-glucose deprivation by inhibiting reactive oxygen species (ROS) generation, and by stabilizing mitochondrial integrity and transmembrane potential. Besides, ghrelin treatment prevents cytochrome *c* release and inhibits caspase-3 activation [108, 109]. Ghrelin also induces the proliferation of neuronal precursor cells in the rat fetal spinal cord [110]. Ghrelin treatment results in adult hippocampal neuronal proliferation in Alzheimer's disease model mice [110]. In general, ghrelin is important for neuronal survival and has a neuronal proliferative and protective role in CNS [101, 111, 112]. This neuroproliferative and neuroprotective role of ghrelin made it a possible target for preparing drugs for neurodegenerative diseases.

### 3.2. Effect of Ghrelin on Gastrointestinal System

The activity of the gastrointestinal tract (GIT) depends on hormones. From those gastrointestinal hormones controlling appetite and food intake, only ghrelin has orexigenic action and is thought to be deeply involved in appetite regulation [113]. All parts of the GIT have an equal distribution of ghrelin receptor [114]. Ghrelin controls appetite, acid secretion which is mediated by histamine [113, 115], and gastric motility via the brain-gut axis [114]. Ghrelin also contributes to the cytoprotection of hepatocytes during hepatic ischemia/reperfusion-induced injury in mice. Ghrelin pretreatment significantly reduces plasma levels of alanine aminotransferase and lactate dehydrogenase, which are markers of hepatic injury [116].

#### 3.2.1. Motility and Secretion

Ghrelin stimulates gastric acid and digestive enzymes secretion in the GIT mainly in the stomach, intestine, and pancreas [117]. IV administration of ghrelin stimulates gastrin [118] and gastric acid secretion [117, 119]. Intraduodenal infusion of ghrelin has been found to increase CCK secretion [120] and pancreatic enzyme secretion [120]. Peripheral injection of ghrelin in healthy humans leads to a significant increase in pancreatic peptide levels [121]. However, other studies on rats showed that ghrelin is a potent inhibitor of pancreatic exocrine secretion in vivo and in pancreatic lobules in vitro by acting indirectly on intrapancreatic neurons. Ghrelin blocks potassium-induced amylase release from pancreatic lobules in vitro and significantly inhibits CCK-stimulated pancreatic secretion in vivo [122]. Ghrelin infusion significantly suppressed C-peptide levels in gastrectomized humans [123]. GOAT inhibitor decreases the acylated ghrelin level and H^+^–K^+^-ATPase activity in vitro [4]. IV or intracerebroventricular (ICV) administration of ghrelin affects not only gastrointestinal secretion but also motility [117]. Endogenous or peripheral administration of ghrelin to humans promotes gastric and small intestinal motility by stimulation of enteric cholinergic neurons with an additional role of serotonin [124–126]. Ghrelin, like motilin, promotes gastric emptying. Colonic motility is activated by ghrelin only when it is administered centrally [127].

#### 3.2.2. Appetite Regulation

Ghrelin reaches a peak level during fasting periods, which intensifies hunger. Its level immediately falls following a meal and satiety developed [128]. Ghrelin is secreted and transported by the blood, binds to GHSR1a on the vagal afferent terminals, and sends information to the central nervous system (CNS) [128]. Then ghrelin inhibits the electrical activity of the efferent vagus nerve to send hunger signals to the CNS. In the hypothalamus, ghrelin acts on the ARC, PVN, and dorsomedial region through the afferent vagus nerve via the nucleus tractus solitarius and activates neuropeptide Y (NPY)/AGRP neurons present in the ARC. Eventually, neuronal pathways relating to feeding are stimulated, and appetite is increased. The ARC of the hypothalamus is the main site of ghrelin's activity in the CNS [129]. Des-acyl ghrelin directly inhibits the ARC in a ghrelin receptor-independent manner to impair the orexigenic effect of ghrelin [130]. A recent study showed that oral ghrelin receptor agonist (z-505) attenuates anorexia after total gastrectomy in rats [131]. This has a therapeutic role for patients with gastric cancer who have undergone gastrectomy and are suffering from anorexia.

#### 3.2.3. Weight Gain Regulation and Energy Balance

Ghrelin targets the hypothalamus and brain stem nuclei to increase appetite and decrease energy expenditure [132] and promote the use of carbohydrates as a source of fuel while sparing fat to increases body weight [114]. ICV, IV, or subcutaneous (SC) administration of ghrelin to humans increases food intake up to 30%, only meal number not meal size [73], and body weight [133]. A study on laboratory mice showed that anti-ghrelin antibodies increase energy expenditure [134]. This demonstrates the role of ghrelin in balancing energy by both increasing caloric intake and declining energy consumption [135]. Ghrelin receptor is an important regulator of thermogenesis [134]. Ghrelin signaling via this receptor decreases thermogenesis to reduce energy expenditure [136, 137]. In addition to decreased thermogenesis, ghrelin also decreases energy expenditure by decreasing locomotor activity [138] and reducing the activity of the sympathetic nervous system (SNS), especially in brown adipose tissue (BAT) [139]. A study on young healthy women shows that ghrelin lowers energy expenditure [140]. Systemic administration of recombinant proghrelin in mice stimulates food intake in the light cycle by acting on unidentified receptor distinct from GHSR1a but not weight gain. It decreases respiratory quotient, indicating an increase in fat consumption and energy expenditure, which is contrary to the effect of acyl ghrelin [141].

In rats, ghrelin stimulates differentiation of preadipocytes, adipogenesis, inhibits adipocyte apoptosis, and antagonizes lipolysis [142]. Ghrelin induces body weight gain by increasing adiposity [60, 133] in a feeding-independent manner [143]. A body weight remission after bariatric surgery was associated with decreased levels of ghrelin [144]. Electrical stimulation of the vagus nerve [145] and antagonists of the ghrelin receptor may be considered as one possible solution to prevent obesity [146]. A recent study showed that the melanocortin 2 receptor accessory protein 2 altered GHSR1a signaling by inhibiting its constitutive activity, as well as by enhancing its G-protein–dependent signaling and blocking the recruitment and signaling of *β*-arrestin in response to ghrelin [147]. This has a therapeutic value for the treatment of obesity. Ghrelin regulates energy balance in the short term via induction of appetite and in the long term via increasing body weight and adiposity [60]. In starvation, ghrelin levels are high, acting as a stimulator of energy intake and inhibitor of energy expenditure and, at the same time, spending calories from carbohydrate intake and stimulating the release of glucose from hepatocytes [7].

*(1) Effect of Ghrelin on Lipid Metabolism*. Understanding the molecular mechanism underlying effects of ghrelin on lipid metabolism will provide new strategies for the design and development of suitable drugs for the treatment of obesity and its comorbidities [148]. Ghrelin promotes adiposity by the activation of hypothalamic orexigenic neurons, stimulates the expression of fat storage-related proteins, and increases lipogenesis and triglyceride uptake in adipocytes mainly in white adipose tissue (WAT) [149]. Furthermore, ghrelin exerts direct peripheral effects on lipid metabolism, including an increase in WAT and stimulation of lipogenesis in the liver through specific pathways in the CNS that are directly connected to WAT, BAT, and liver, thereby directly influencing adipocyte and hepatic metabolism [150].

**Figure 8:**
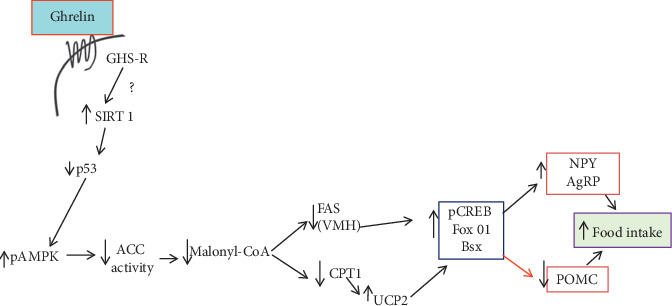
The CNS ghrelin modulates hypothalamic fatty acid metabolism by activating sirtuin 1 (SIRT1) and AMPK, which in turn stimulates transcription factors essential for NPY/AgRP, which finally affects food intake. The hypothetical molecular step which has not been described is indicated by the red arrow. The question mark indicates a black box in the molecular events triggered after the activation of the GHSR1 and before sirtuin 1. UCP2: uncoupling protein 2; pCREB: phosphorylated cAMP response element-binding protein; FOXO1: forkhead box O1; NPY: neuropeptide Y; AgRP: agouti-related peptide [161].

*(2) Effect of Ghrelin on Central/Hypothalamic Lipid Metabolism*. Hypothalamic nuclei including ARC, PVN, dorsomedial, and ventromedial (VMH) nuclei contain high levels of key enzymes modulating lipid metabolism, such as AMP-activated protein kinase (AMPK), acetyl-CoA carboxylase (ACC), carnitine palmitoyltransferase 1 (CPT1), fatty acid synthase (FAS), and malonyl-CoA decarboxylase [151]. Fasting reduces the production of hypothalamic malonyl-CoA [152], shifting metabolic substrate utilization away from glycolysis and toward lipid oxidation [153]. Malonyl-CoA acts indirectly on CPT1 and thus prevents the access of long-chain fatty acyl-CoA to the mitochondria, which would decrease food intake [154, 155]. Hypothalamic fatty acid metabolism mediates the orexigenic effect of ghrelin [156, 157]. Ghrelin-induced food intake activates hypothalamic sirtuin 1 (SIRT-1), which deacetylates p53 and thereby activates AMPK [158]. The activated AMPK subsequently inhibits fatty acids synthesis, leading to lower hypothalamic levels of malonyl-CoA and increased CPT1 activity [159]. The hypothalamic fatty acid oxidation pathway modulated by AMPK, together with the decrease of FAS expression in the VMH and the activation of CPT1, leads to changes in hypothalamic mitochondrial respiration and production of ROS in mice, which are dependent on uncoupling protein 2 [160] (Figure 8).

**Figure 9:**
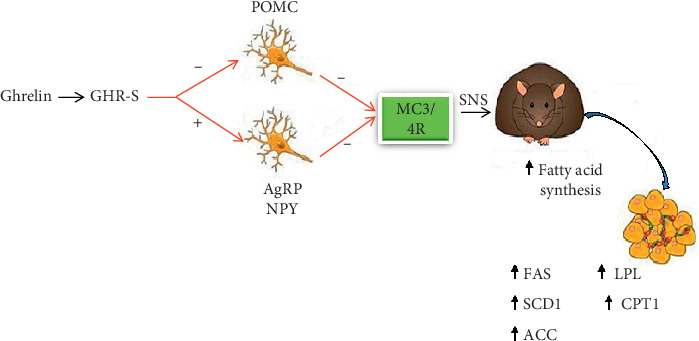
CNS ghrelin increases adiposity by favoring peripheral lipid deposition. Ghrelin binds to its receptor and hypothalamic neuropeptides (NPY/AgRP vs. POMC), and thereby melanocortin receptors are likely involved in the lipogenic action of ghrelin. Red arrows indicate the hypothetical molecular steps which have not been described for the lipogenic action of ghrelin. POMC: proopiomelanocortin; SCD1: stearoyl-CoA desaturase-1 [161].

*(3) Effect of Ghrelin on Peripheral Lipid Metabolism*. Peripheral lipid metabolism is regulated by central ghrelin mainly in a GH-independent manner. The central effects of ghrelin on adipocyte metabolism are direct by stimulating lipogenesis in WAT via SNS independently of food intake [149]. The effect of ghrelin on adipose tissue is mediated by the autonomic nervous system (ANS). Acute injection of ghrelin into the third cerebral ventricle decreases SNS activity in BAT [162]. On the other hand, chronic central administration of ghrelin to GH-deficient rats increases body fat by increasing protein expression, mRNA, and lipogenic enzyme expression in WAT including stearoyl-CoA desaturase-1, FAS, ACC, and lipoprotein lipase [149], while it reduces expression of the fat-oxidation promoting CPT1 in the WAT of rats [148] (Figure 9). On the contrary, hepatic lipogenesis de novo is regulated by central ghrelin in a GH-independent manner whereas hepatic lipid mobilization by ghrelin is GH dependent. This was evidenced by a study on normal and GH-deficient rats which showed that the activity of CPT1, the key enzyme modulating fatty acid oxidation/mobilization, is enhanced after central ghrelin infusion in a GH-independent fashion in WAT. However, activation of the central ghrelin system specifically decreases hepatic CPT1 activity in normal rats but not the liver of dwarf rats, suggesting that ghrelin needs GH to effectively decrease CPT1 [148, 149]. These central effects of ghrelin on adipocyte metabolism are direct by stimulating lipogenesis in WAT via SNS, independently of food intake [149].

Ghrelin activates its receptor on hepatocytes to promote lipogenesis via a mechanism involving the mTOR-proliferator-activated receptor-*γ* (PPAR*γ*) signaling pathway. The blockage of its receptor or removal of its gene suppresses de novo lipogenesis which in turn helps to prevent and treat obesity-associated hepatic steatosis. Furthermore, the stimulatory effect of ghrelin on hepatic lipogenesis was significantly attenuated by PPAR*γ* antagonism in cultured hepatocytes and PPAR*γ* gene-deficient mice. This gives insight for the treatment of nonalcoholic fatty liver disease via targeting hepatic ghrelin receptor/mTOR/PPAR*γ* [163]. Ghrelin stimulates lipogenesis and decreases fatty acid oxidation in the liver by directly activating its receptor on hepatocytes [163, 164].

Both isoforms of ghrelin, acyl ghrelin and des-acyl ghrelin, pointedly increase triglycerides content in rat hepatocytes. IV infusion of ghrelin also increases triglycerides, cholesterol, and free fatty acid levels in rats [165]. Cells overexpressing ghrelin, 3T3-L1 cells, inhibit the differentiation of preadipocytes into adipocytes. Both ghrelin overexpression and exogenous ghrelin stimulate cell proliferation via acting on a novel unidentified ghrelin receptor subtype. This cell proliferation results in inhibition of adipogenesis [166].

Ghrelin also increases fat oxidation in muscle. A study on isolated, mature skeletal muscle obtained from male rats showed that ghrelin directly stimulates fatty acid oxidation in oxidative and glycolytic muscle. Ghrelin dampens epinephrine-induced lipolysis in oxidative muscle. Both ghrelin isoforms stimulate fatty acid oxidation in skeletal muscle possibly by phosphorylating AMPK and its downstream effector, ACC, which in turn relieves malonyl-CoA inhibition on mitochondrial CPT1. This increase in fatty acids oxidation ranged from approximately 15 to 42% and was accompanied by increases in ACC phosphorylation, a downstream target of AMPK [167].

### 3.3. Effect of Ghrelin on Cardiovascular System

Ghrelin has a variety of cardiovascular activities in both physiological and pathophysiological states. It affects heart, blood vessels, and blood volume [168]. Ghrelin receptors are present throughout the heart. Different studies have proved that ghrelin has strong cardiovascular protective effects [169, 170]. It is associated with anti-inflammatory effects, inhibition of atherosclerotic plaque formation, and plaque stability in the cardiovascular system [171].

#### 3.3.1. Effect of Ghrelin on Heart

Ghrelin is important for the improvement of cardiac performance by regulating intracellular calcium concentration [172]. In humans both SC and IV administration of ghrelin increase cardiac output, but the mechanism is different: SC administration increases left ventricular contractility which leads to an increased ejection fraction [173, 174], whereas IV injection increases cardiac output by decreasing left ventricular afterload [175]. This decrease in afterload may be a result of a central effect of ghrelin on the nucleus of the solitary tract [175, 176] and its potent vasodilator effect [177]. Ghrelin has also an effect on the electrical activity of the heart by suppressing cardiac sympathetic nerve activity [100] and stimulating cardiac parasympathetic nerve activity [100, 178]. Besides, ghrelin provides a protective role for the heart, by inhibiting cardiomyocyte apoptosis, reducing fibrosis, and improving cardiac function [169, 170, 179]. After myocardial infarction, exogenous administration of ghrelin preserves cardiac function [169] possibly by the promotion of angiogenesis [180] and by its anti-inflammatory effects and protection of oxidative damage [169]. Des-acyl ghrelin also protects the heart against cardiac dysfunction by inhibiting excessive collagen deposition [181].

Ghrelin reduces the incidence of fatal arrhythmias and ventricular remodeling, leading to improvements in heart failure [169]. Overall ghrelin has a protective role in cardiovascular problems [182]. These effects of ghrelin are good news for treating ischemic heart disease, which is the leading cause of cardiovascular morbidity and mortality worldwide [183], and other heart diseases including myocardial infarction.

#### 3.3.2. Effect of Ghrelin on Blood Vessel

Ghrelin has vasodilator influences leading to a decrease in mean arterial pressure without changing the heart rate in healthy humans [175]. It inhibits atherosclerotic plaque formation and promotes plaque stability [171, 184]. A study done among humans showed that ghrelin protects the blood vessels by inhibiting the vascular endothelial cell apoptosis, improves endothelial dysfunction, suppresses vascular inflammation, and enhances endothelial nitric oxide synthase (eNOS) expression [185, 186]. Ghrelin causes vasodilation either through NO-independent mechanisms, by inhibiting SNS resulting in low blood levels of NE contributing to the vasodilation effect of ghrelin [187], or through NO-dependent manner. Ghrelin activates eNOS, through GHSR-mediated Akt and AMPK signal pathways, by rapidly inducing eNOS phosphorylation on Ser-1177 in cultured endothelial cells and in intact vessels resulting in an acute increase in NO production that is involved in ghrelin anti-inflammatory effects. Akt and AMPK are the major mediators for ghrelin activation of eNOS both in vitro and in vivo. Ghrelin-activated Akt is involved in eNOS phosphorylation and NO production [188, 189]. Ghrelin induces Akt phosphorylation on active site Ser-47 and thereby stimulates eNOS activation and the consequent NO production. Ghrelin also activates AMPK, by phosphorylating it on the activation site Thr-172, which plays a critical role in ghrelin activation of eNOS. AMPK can also phosphorylate directly eNOS at Ser-1177 [190–192]. Ghrelin receptor/G-protein/calcium-dependent pathway mediates activation of AMPK, Akt, eNOS, and NO production. In response to ghrelin, calcium/calmodulin-dependent protein kinase 2 (CaMKKs), in particular, its *β*-isoform (CaMKK*β*), is involved in AMPK activation [193], and it directly activates Akt [194]. Once NO is produced, it causes vasodilation by three different major signaling pathways: (1) NO stimulates soluble guanylyl cyclase in the vascular smooth muscle cells to induce the formation of cyclic guanosine monophosphate (cGMP). CGMP activates protein kinase G (PKG). CGMP-dependent activation of PKG I leads to phosphorylation of different membrane proteins in the sarcoplasmic reticulum. PKG I phosphorylates phospholamban that activates sarcoplasmic reticulum ATPase (SERCA), which in turn leads to sequestration of Ca^2+^. (2) activation of PKG I leads to phosphorylation of a protein called inositol 1,4,5-trisphosphate (IP3) receptor–associated cGMP kinase substrate (IRAG) [195]. Phosphorylation of IRAG results in a strong inhibition of IP_3_-evoked Ca^2+^ release from the sarcoplasmic reticulum. (3) NO activates Ca^2+^-dependent K^+^ channels, increases the outward potassium current [196], and causes hyperpolarization of the membrane. This hyperpolarization inhibits Ca^2+^ entry. These 3 mechanisms result in low intracellular Ca^2+^ concentration. The reduction of the intracellular Ca^2+^ concentration reduces the formation of the Ca^2+^-calmodulin–myosin light chain kinase complex and inhibits vasoconstriction. Protein kinase G also acts on sarcoplasmic reticulum calcium ATPase to promote the reuptake of cytosolic calcium into the sarcoplasmic reticulum. This leads to the decrement of intracellular calcium concentration resulting in inactivation of calmodulin, which is no longer able to activate myosin light chain kinase. Calcium depletion also increases the activity of myosin light chain phosphatase. The actin-myosin cross-bridge is broken and smooth muscle relaxation ensues to cause vasodilation [197]. Therefore, the identification of ghrelin regulatory pathways on eNOS activation may give insights about the therapeutic potential of ghrelin to correct endothelial dysfunction in patients with cardiovascular disease and diabetes [198].

Ghrelin is also a potent inhibitor of sulfur-containing amino acid production called homocysteine, one of the major causes of endothelial dysfunction that decreases endothelium-dependent vasorelaxation and eNOS reactivity [199, 200]. This information will provide a good opportunity to treat patients with atherosclerosis.

*(1) Blood Pressure.* Ghrelin receptors that are highly expressed in the heart, kidneys, and blood vessels are important for blood pressure regulation. The concentration of circulatory ghrelin is inversely correlated with arterial blood pressure (ABP) and was found to be low in hypertension [201, 202]. Ghrelin regulates blood pressure by a short-term mechanism including modulation of the ANS and direct vasodilator activities and long-term mechanisms by kidney diuresis [178, 203]. Ghrelin infusion is reported to lower blood pressure by promoting peripheral vasodilatation via both nitric NO-dependent and NO-independent mechanisms [173]. A long-term ghrelin treatment for salt-induced hypertension in Dahl rats decreased ABP by significantly increasing urine output and Na^+^ excretion [203]. Acyl ghrelin and combined acyl ghrelin and des-acyl ghrelin infusions decreased systolic blood pressure, diastolic blood pressure, mean ABP, heart rate, and temperature through modulation of ANS [204]. Acute administration of acyl, but not des-acyl ghrelin, decreases blood pressure in healthy humans [204]. These findings provide basic insight into the clinical application of ghrelin or its derivatives by modulating its signaling pathway to treat patients with hypertension [203].

### 3.4. Effect of Ghrelin on the Immune System

**Figure 10:**
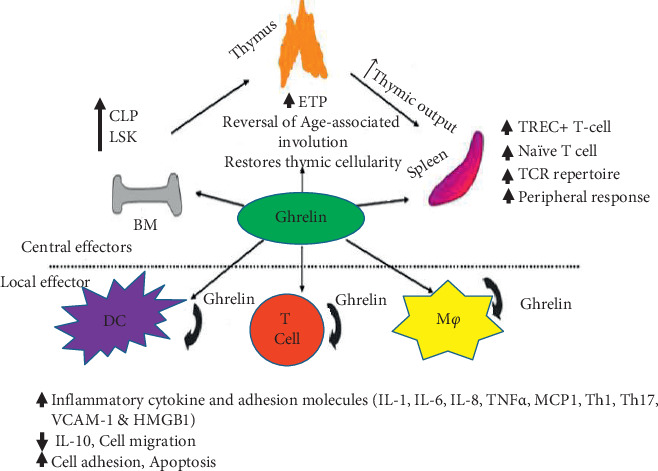
Biological impact and therapeutic use of ghrelin on immune cells and in various inflammatory disease states [216]. VCAM: vascular cell adhesion molecule; HMGB: high mobility group protein 1; MCP1: monocyte chemoattractant protein 1; CLP: common lymphoid progenitors; DC: dendritic cell; M*φ*: macrophage; LSK: Lin^−^Sca-1^+^c-Kit^+^; ETP: early thymocyte progenitors; TREC^+^-: T-cell receptor excision circle.

Ghrelin appears as a natural antimicrobial and anti-inflammatory peptide, widely distributed in all body tissues and especially abundant in nonspecific immune organs (physical barriers) such as oral cavity, stomach, gut, and skin providing protection role for innate immunity and response against infections [205–207]. Ghrelin has been shown to regulate the organism's immune function [208, 209] and to have anti-inflammatory effects [210–212] by acting mainly on the innate and adaptive immune systems. The anti-inflammatory effects of ghrelin are observed in immune cells of both myeloid and lymphoid lineages [213]. Ghrelin acts on human T lymphocytes and monocytes via GHSR to specifically inhibit the mRNA and protein expression of inflammatory cytokines such as IL-1*β*, IL-6, and TNF-*α*, enhancing the expression of anti-inflammatory cytokine IL-10 and inhibiting apoptosis of immune cells [210, 214, 215] (Figure 10). It is also shown that ghrelin suppresses inflammation in several disease models by attenuating neutrophil migration and promoting phagocytosis of an apoptotic neutrophil by macrophages [216]. However, another study on humans reported that ghrelin does not modulate neutrophil in vitro [217]. Ghrelin improves tissue perfusion and function in severe sepsis via downregulation of endothelin-1 [218, 219]. Human ghrelin also plays an important role in reestablishing the proliferation of CD4 T cells and serves as a promising therapeutic agent in sepsis [220].

Moreover, ghrelin has also been shown to promote lymphocyte development in the primary lymphoid organs (bone marrow and thymus) and to ablate age-associated thymic involution [216]. Ghrelin inhibits apoptosis [221] and promotes thymopoiesis during aging, providing an opportunity to prepare therapeutics to induce thymic function in immunocompromised subjects [222]. Generally, ghrelin is a potent anti-inflammatory mediator both in vitro and in vivo, and it is a hopeful therapeutic agent in the treatment of both acute and chronic inflammatory diseases and injury.

### 3.5. Effect of Ghrelin on Musculoskeletal System

Ghrelin induces beneficial effects on muscle strength and energy metabolism via a GH-dependent mechanism. Ghrelin prevents tumor- and cisplatin- (a chemotherapeutic agent) induced muscle wasting. Ghrelin prevents muscle atrophy by downregulating inflammation [223]. This role of ghrelin is important for the prevention of cachexia which is a complication of many chronic diseases [223, 224]. Ghrelin affects the three types of muscle: skeletal, cardiac, and smooth muscle cells.

#### 3.5.1. Skeletal Muscle

Ghrelin has an effect on the excitation of skeletal muscle. It acts on its receptor coupled to G-protein and activates a phospholipase C-signaling pathway producing inositol triphosphate (IP3) and diacylglycerol (DAG) [177]. Both IP3 and DAG produce a persistent increase in the Ca^2+^ levels that will stimulate the protein kinase C (PKC). PKC produces phosphorylation of the Cl^−^ and K^+^ channels to decrease chloride and potassium conductivity [177, 225], which in turn makes the muscle fibers less negative and easily excitable to initiate contraction.

Sustained acyl ghrelin administration enhances muscle mitochondrial oxidative capacity [226] by increasing food intake, hepatic gluconeogenesis, and fat deposition in rats [227]. Enhancement of mitochondrial oxidative capacity ensures the production of more ATP and helps the muscle to resist fatigue. Furthermore, ghrelin increased the number of fatigue resistant, oxidative (type IIa) muscle fibers, preventing the decline in muscle strength and endurance seen with aging [227].

Des-acyl ghrelin fosters muscle regeneration by promoting myoblast differentiation and regeneration [228]. Both ghrelin and des-acyl ghrelin stimulate proliferating C2C12 skeletal myoblasts [229]. Ghrelin is also important for skeletal muscle cell regeneration following injury, which depends on satellite cells, quiescent precursors that activate, proliferate, and differentiate to repair the damaged tissue [228]. Des-acyl ghrelin reduces skeletal muscle mitochondrial ROS generation [230] and ROS-induced cell injuries by inducing the expression of superoxide dismutase-2 in satellite cells resulting in the induction of the myogenic process and reduction of functional impairment [231]. Administration of high doses of ghrelin analogs significantly reduces myostatin, a member of the transforming growth factor-*β* superfamily, considered as a negative regulator of muscle growth [224]. Moreover, ghrelin inhibits muscle and protein catabolism [224].

#### 3.5.2. Smooth and Cardiac Muscles

Ghrelin improves cardiac contractility in pathological cardiac conditions [177]. Chronic subcutaneous administration of ghrelin improves cardiac performance by reduction of the potassium conductivity in rats with heart failure [232]. Activation of GHSR1a by ghrelin stimulates a G-protein that activates the PLC-signaling pathway producing IP3 and DAG. Both IP3 and DAG lead to an elevation of myocardial Ca^2+^ levels via stimulation of Ca^2+^ influx through the voltage-gated Ca^2+^ channel and Ca^2+^ release from the sarcoplasmic reticulum; then it will improve cardiac contractility [177]. It has been also shown that repeated intravenous administration of ghrelin improves left ventricular function by increasing muscle strength [233]. However, ghrelin has negative inotropic and lusitropic effects in vitro [177]. This negative lusitropic effect, a slower rate and an earlier onset of myocardial relaxation, is modulated by prostaglandins and NO [234]. Ghrelin has an antiapoptotic effect in cardiomyocytes [177]. Regarding smooth muscle, ghrelin modulates vascular tone and increases gut transit. It has a potent vasodilator and systemic hypotensive effect. This effect is due to the stimulation of opening K^+^ and Cl^−^ channels (to make the cell more negative) and release of endothelial-derived relaxing factor which leads to relaxation of the vascular smooth muscle [235]. Regarding its effect on intraocular eye muscles, it relaxed circular muscle of iris by inducing the release of prostaglandins, resulting in pupillary dilation [177].

#### 3.5.3. Bone

Like many parts of the body, ghrelin and its receptor are expressed in osteoblast cells [136]. Different studies showed that ghrelin increases bone mineral density by stimulating osteoblast cells [224, 236–238]. Ghrelin also regulates bone formation by activating phosphorylation of AMP-activated protein kinase (AMPK). Leptin and ghrelin have opposite effects on bone. Ghrelin treatment activates osteoblasts but has no effect on osteoclasts [239]. Ghrelin also increases bone mass independently of food intake or weight gain [240].

### 3.6. Effect of Ghrelin on the Respiratory System

During intrauterine life, the developing lung seems to be a major source of ghrelin with decreasing levels of expression throughout gestation. The developing lung and pancreas express ghrelin earlier than other organs. Significant ghrelin expression during 7–18 weeks of gestation was reported, suggesting that ghrelin might act as a regulator of fetal lung development by autocrine/paracrine mechanisms [14]. A recent study done on adult rats demonstrated that ghrelin modulates pulmonary vascular remodeling and hypertension [241]. Ghrelin produced in the lung may represent one of the major factors responsible for the mid-gestational peak of GH, hence modulating fetal lung development. Although ghrelin has an important role in the development of fetal lung, ghrelin knockout mice do not show significant lung abnormalities [242]. Ghrelin treatment for hypoxic animals reduced the overexpression of hypoxia-induced expression of protein kinase C-*ε* and PKC-*δ*, cause of pulmonary vasoconstriction, and improved the hypoxic pulmonary vasoconstriction [243]. It is also shown that the administration of ghrelin reduced lung injury in a rat model of ventilation-induced injury. This finding has a therapeutic advantage for patients who are in the intensive care unit and at high risk of ventilation-induced lung injury [244].

### 3.7. Effect of Ghrelin on Renal System

Ghrelin has a protective role in the kidney. Ghrelin administration to rats prevented tissue damage in obstructive uropathy cases [245]. Acyl and des-acyl ghrelin have strong potential to improve chronic kidney disease [246]. Ghrelin also has been shown to exert potential protective effects on the kidney ischemia-reperfusion injury and subsequent kidney dysfunctions through inhibition of oxidative stress and apoptosis and modulation of inflammation [247]. Ghrelin inhibited renal fibrosis by attenuating the production of collagen, deposition of extracellular matrix (ECM), and fibronectin. Ghrelin has therapeutic potential for patients with obstructive nephropathy [248]. Ghrelin protects the kidney from cisplatin-induced nephrotoxicity in mice through inhibition of inflammatory reactions [249]. The same protection role of ghrelin is also reported on other studies done on mice with sepsis. The vagus nerve could play an important role in the renal protective effects [250]. A recent study also showed that ghrelin has potential protective role during the septic process [251]. This role of ghrelin creates opportunity to prepare a therapy from it for treating life threatening septic diseases and shock.

### 3.8. Effect of Ghrelin on Reproductive System

Reproduction is a hormone-dependent process under the effect of gonadotropin-releasing hormone (GnRH). The onset of puberty is dependent on activation of the GnRH pulse generator, which is regulated by multiple peripheral and central peptides including ghrelin. Food intake and adipose tissue accumulation and their impacts on the GnRH secretion are mediated through ghrelin and leptin [252]. Ghrelin has been implicated in modulating reproductive function, acting at all levels of the hypothalamic-pituitary-gonadal (HPG) axis [253]. Ghrelin delays pubertal onset both in male and female rats, with males appearing to be more sensitive than females [253]. Several studies have indicated that ghrelin has an inhibitory effect on gonadotropin pulsatility, which is involved in the regulation of puberty onset and may regulate spermatogenesis, follicular development, and ovarian cell functions in humans [254]. Acyl ghrelin exerts an inhibitory effect on follicle-stimulating hormone (FSH) and luteinizing hormone (LH) by acting on its receptor on GnRH neurons, whereas it promotes prolactin secretion by its direct action on pituitary somatomammotropin cell [17, 252, 255, 256]. Des-acyl ghrelin may exert some of the central effects through the additional, not yet proved receptor including the gonadotropin-inhibitory hormone G-protein-coupled (GPR) receptor 147 (GnIH-GPR147) system by affecting kisspeptin-GnIH-GnRH pathway [252]. A study done using ovariectomized rats demonstrated that ghrelin administration significantly reduces LH pulsatility and suppresses kisspeptin mRNA expression [257]. Ghrelin acts on the anterior pituitary gland to suppress the release of FSH and LH [252, 258]. Ghrelin suppresses LH secretion in vivo and decreases LH responsiveness to GnRH in vitro [259]. A recent study on mice showed that appropriate levels of acyl and des-acyl ghrelin are necessary for optimal ovarian maturation but its absence cannot prevent its success [6]. In women, ghrelin enhances the proliferation of ovarian cells, whereas in men it counters Leydig cell functions [17]. Another study done on humans show that acyl ghrelin inhibits steroid biosynthesis by ovarian granulosa lutein cells [260]. On the other hand, the level of GHSR was found low in the endometrium of infertile women [261], which implies that a balanced level of ghrelin system is important for healthy reproduction. A recent study shows that ghrelin administration significantly decreases testosterone plasma levels and impairs spermatogenesis possibly by inhibition of the hypothalamic-pituitary-gonadal axis [262]. The majority of studies report that ghrelin inhibits testosterone production and spermatogenesis. The appropriate concentration of ghrelin is found to be important in early gestational events. In a study on mice, both administrations of a high dose of acyl ghrelin and GHSR antagonism during peri-implantation and early gestation impair fertilization, implantation, and embryo development [260]. Ghrelin has also an important role in fetal and neonatal energy balance and in allowing fetal adaptation to an adverse intrauterine environment [263].

#### 3.8.1. Effect of Ghrelin on Sexual Behavior

Ghrelin receptor signaling is important for the full expression of appetitive sex behavior, and it is shown that ghrelin plays an important role in male sexual behavior. Central ghrelin receptor stimulation modulates sex motivation in male rats in a site-dependent manner. Within the VTA, ghrelin may act to enhance sex motivation, while acting on the medial preoptic area (mPOA) to decrease sex motivation and promote foraging [264]. Another study on mice showed that genetic suppression of the GHSR1a in male mice attenuates the preference for female mice, sexual motivation, and interaction, by effects on DA neurotransmission [72]. After the hypothalamic ghrelin administration, the reproductive performance of male mice was found to decrease [262].

#### 3.8.2. Effect of Sex on Ghrelin Level and Action

Different studies have shown that gastric ghrelin cells and serum ghrelin levels were significantly higher in females than males indicating that secretion of ghrelin can be under control of sex hormones [265]. A study using isolated stomach cells found that estrogen treatment significantly stimulates ghrelin mRNA expression and the number of immunopositive cells for ghrelin [266]. Similarly, other studies also reported that estrogen upregulates plasma ghrelin level [267]. Nevertheless, postmenopausal estrogen-replacement therapy increases total and acyl ghrelin level slightly [268]. Another study on peripubertal children showed that pharmacological increase in sex hormones is associated with a marked decline in circulating levels of ghrelin in boys but not girls [269]. Inconsistently, in a study on pregnant rats [270] and normal pregnant women [271], a significant decrease in plasma ghrelin levels was observed, which suggests that increased estrogen levels directly induce a downregulation of ghrelin expression. This finding is not in line with the aforementioned studies that support the idea that estrogen stimulates ghrelin level. It may be due to the presence of pregnancy-related factors other than the raised level of estrogen. It has been also shown that sex hormones have effects on the ghrelin level in pathological conditions. Testosterone therapy to hypogonadal men increases plasma ghrelin levels markedly [272]. In the case of women with polycystic ovary syndrome, the high androgen levels associated with this syndrome suppressed ghrelin levels [273, 274]. Sex hormones have also an effect on the action of ghrelin. A study on ovariectomized female and male rats shows that estradiol diminishes the orexigenic action of ghrelin [275]. Ovariectomy increases food intake, by releasing ghrelin due to a tonic inhibitory effect of estradiol, and leads to ghrelin mediated weight gain [275]. Overall, there is controversy over the effect of estrogen on ghrelin levels. Therefore, further studies are needed to confirm the effect of sex hormone on ghrelin.

### 3.9. Effect of Ghrelin on Endocrine System

One of the first known functions of ghrelin following its discovery is the stimulation of GH release. Ghrelin stimulates the release of GH by having a synergistic effect with growth hormone releasing hormone (GHRH) and indirectly by inhibiting growth hormone inhibitory hormone [7, 58, 59]. Plasma GH and pituitary GH mRNA levels were significantly increased in the animals injected with ghrelin [276]. Ghrelin modulates lactotrophs and corticotropic activity and stimulates the release of PRL, adrenocorticotropic hormone (ACTH), and cortisol secretion. Ghrelin also affects the secretion of vasopressin and oxytocin. ICV or IV administration of ghrelin stimulates release of vasopressin and oxytocin in cell cultures of neurohypophyseal tissue [277]. Ghrelin receptor antagonists can block the enhancement of vasopressin and oxytocin secretion induced by ghrelin [278].

Moreover, ghrelin controls insulin secretion and influences thyroid function [58, 279, 280]. Acyl ghrelin regulates the stress response by acting indirectly on corticotropin-releasing hormone (CRH) neurons in the paraventricular nucleus and directly at the anterior pituitary gland to facilitate ACTH release and to increases circulating ACTH to avoid mood disorder caused by its imbalance [218, 281].

#### 3.9.1. Effect of Ghrelin on Pancreas and Glucose Homeostasis

Ghrelin affects both the exocrine and endocrine functions of the liver [7]. In humans, ghrelin decreases insulin secretion [280, 282]. Ghrelin via GHSR directly stimulates glucagon secretion in pancreatic *α*-cells [283]. Ablation of ghrelin, GHSR, or GOAT enhances insulin release [7]. This insulin static action of the ghrelin/GHSR system could optimize the amount of insulin released on systemic demand [7]. The ghrelin blockade counteracts the obesity-associated glucose intolerance [58]. Ghrelin deletion in obese mice decreases hyperglycemia and enhances glucose-induced insulin secretion, thereby improving insulin sensitivity in peripheral tissues [284].

Ghrelin receptor antagonism might be of therapeutic value to improve blood glucose level in type 2 diabetes [285]. In fatty acid-binding protein-ghrelin transgenic mice, an increase in plasma concentration of des-acyl ghrelin was found and the glucose level was significantly lower than controls following glucose administration. These mice had a greater hypoglycemic response to insulin administration [286]. This shows its role in improving glucose tolerance and insulin sensitivity.

#### 3.9.2. Effect of Ghrelin on the Thyroid Gland and Thyroid Hormones

Ghrelin plays inhibitory roles in the regulation of thyroid hormones in the PVN [287]. Administration of ghrelin to PVN neuron modulates thyrotropes and decreases rat serum thyroid stimulating hormone (TSH) and tetraiodothyronine (T4) level. In support of this, several in vivo studies on rats show that ghrelin injection causes a decline in thyrotropin-releasing hormone (TRH), TSH, triiodothyronine (T3) hormone, and T4 [13, 288] hormone [289]. Similar studies in humans confirm the inhibitory role of ghrelin on the plasma concentration of TSH [289, 290]. Ghrelin has been shown to have a suppressive impact on thyrocytes [13]. However, other studies in humans showed no effect [291]. This gives a clue to target ghrelin as one possible way to treat patients with thyroid disorder.

### 3.10. Effect of Ghrelin on Integumentary System

Ghrelin also has an effect on the integumentary system (skin). A study on a mouse model showed that ghrelin prevents the development of dermal fibrosis through its antifibrotic action which suggests that ghrelin might be a candidate for research aiming to prepare a drug for the treatment of human scleroderma, a chronic inflammatory disease characterized by widespread fibrosis of the skin [292]. It is also shown that, in rats with combined radiation and burn injury, ghrelin accelerates wound healing [293]. Furthermore, ghrelin affects the skin by stimulating the release of CRH from the hypothalamus [294]. CRH directly stimulates the skin's natural lipids production that is mediated by CRH receptor 1 [18]. A study conducted on seborrheic patients showed that increased ghrelin levels in the blood may induce overexpression of CRH in sebaceous glands, and then CRH plays its role in inducing lipogenesis in sebocytes and the progression of seborrhea [18].

## 4. Ghrelin and Autophagy

Many protective functions of ghrelin in each system mentioned above are mediated in part by a regulation of autophagy that involves multiple physiological processes. Autophagy is an auto-degradative process whereby cytosolic organelles and proteins are compartmentalized within a double-membrane vesicle, termed autophagosome, that translocates to lysosomes for fusion and content degradation, to maintain the cellular quality control or provide an alternative source of energy during starvation [295]. Autophagy has two roles: the first is to degrade bulk cytoplasmic contents, abnormal protein aggregates, and damaged organelles during excess nutrient status; the second is to adjust an alternative source of energy, which is important during nutrient starvation or stress [296]. This autophagy process is inhibited by ghrelin during inflammatory conditions like acute hepatitis, liver fibrosis, or adipose tissue inflammation to prevent further injury. However, under normal conditions ghrelin stimulates autophagy by activating AMP-activated protein kinase in different target organs to regulate lipid and glucose metabolism, remodeling and protection of small intestine mucosa, and protection against cardiac ischemia as well as higher brain functions such as learning and memory [297, 298].

### 4.1. Effect of Ghrelin and Autophagy on the Liver, Adipose Tissue, and Skeletal Muscle

Using ghrelin to induce autophagy is a novel approach to prevent and treat nonalcoholic fatty liver disease, obesity, and type 2 diabetes. Hepatic lipid metabolism is mediated in part by autophagy, which facilitates the breakdown of lipid droplets and its mobilization to lysosomes in a process known as lipophagy [299]. This is evidenced by a study showing that inhibition of autophagy leads to a significant increase in the number of lipid droplets [300]. During energy, depletion acyl ghrelin prevents hypoglycemia through the induction of autophagy [177]. In a disease condition, acyl and, to a lesser extent, des-acyl ghrelin activate autophagy in rat hepatocytes contributing to the improvement of nonalcoholic fatty liver disease [301]. On the other hand, in animal models of acute hepatitis and liver fibrosis, which are associated with liver inflammation, they inhibit hepatic autophagy to avoid further hepatic damage [302].

The autophagy activity in skeletal muscle is stimulated by insulin. Insulin resistance in type 2 diabetes has been linked to suppression of autophagy markers in the skeletal muscle of diabetic mice. Ghrelin induces autophagy in skeletal muscle to improve insulin signaling and apoptosis [303].

### 4.2. Central Effects of Ghrelin by Enhancing Autophagy in Neurons

This has a potential role in age-related neurodegenerative disorder. During aging and in several neurodegenerative diseases including Alzheimer's, Parkinson's, and Huntington's, autophagy is impaired resulting in the presence of misfolded proteins or the accumulation of dysfunctional organelles. Ghrelin activates autophagy in neurons to extend lifespan [304, 305] and to improve cognitive function in experimental models of neurodegenerative diseases [305]. In humans ghrelin restores the impaired ubiquitin-proteasome system and activates autophagy in a cellular model of Alzheimer's disease, favoring the elimination of toxic aggregates [304].

### 4.3. Effects of Ghrelin and Autophagy on Cardiovascular System

Ghrelin induces autophagy in the cardiovascular system to protect against cardiac ischemia, diabetic cardiomyopathy, and vascular calcification. Myocardial cell function is dependent on controlled regulation of protein synthesis, processing, and elimination. This, in turn, depends on autophagy [306]. During acute cardiac ischemia, both ghrelin isoforms, but in particular des-acyl ghrelin, markedly reduce infarction size and preserve cardiac function, in part, by the activation of autophagy to remove dysfunctional mitochondria after myocardial infarction in mice [307]. Intraperitoneal administration of des-acyl ghrelin in obese, diabetic mice protects against diabetic cardiomyopathy by enhancing autophagy [181]. Moreover, chronic intraperitoneal administration of ghrelin improves autophagy in vascular smooth muscle cells from rats with vascular calcification in an AMPK-dependent manner [308].

## 5. Conclusion

In this review, we confirmed that ghrelin has a broad range of physiological effects on all body systems and contributes multitude of functions in physiological and pathological conditions. It is the only known systemic signal to specifically promote food intake and positive energy balance and to facilitate the development of adiposity by decreasing fat oxidation, making it a target for treatment of obesity and obesity-related chronic diseases. It induces gastrointestinal functions and acid secretion. It has a cytoprotective role in the majority of body systems and prevents chronic inflammatory diseases by its anti-inflammatory, antimicrobial, antifibrotic, and antiapoptotic effect. Its protective role is also mediated in part by the regulation of autophagy that involves multiple physiological processes. It improves cardiovascular functions by enhancing cardiac performance and its vasodilator effect. It improves higher brain functions and prevents neurodegenerative diseases through its neuroproliferative and protective role. It enhances sensory function and sleep and has analgesic role. Ghrelin has effect on both endocrine and exocrine glands and has a stimulatory effect on secretion of most hormones of the endocrine system. Ghrelin has an inhibitory effect on the sympathetic nervous system and the immune system. Having a clear understanding of the ghrelin effect in each system has therapeutic implications. Future studies are necessary to elucidate the molecular mechanisms of ghrelin actions as well as its application as a GHSR agonist to treat most common diseases in each system without any paradoxical outcomes on the other systems.

## Abbreviations

ACTH: Adrenocorticotropic hormone
ABP: Arterial blood pressure
CNS: Central nervous system
CRH: Corticotrophin releasing hormone
GHSR: Growth hormone secretagogue receptor
IV: Intravenous
PVN: Paraventricular nucleus
SC: Subcutaneous
VTA: Ventral tegmental area.
